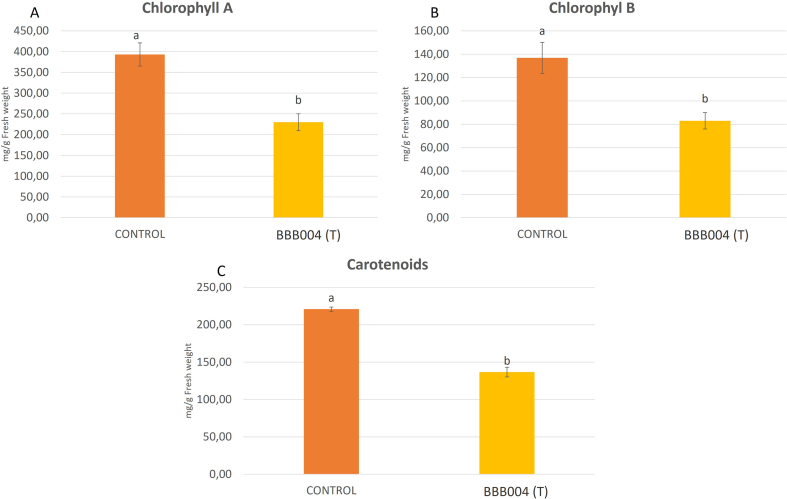# *Peribacillus aracenensis* sp.nov., a plant growth promoting bacteria for agriculture in water-scarce conditions isolated from *Pinus pinaster* rhizosphere

**DOI:** 10.1016/j.heliyon.2024.e39973

**Published:** 2024-11-05

**Authors:** Enrique Gutierrez-Albanchez, Ana García-Villaraco, José Antonio Lucas, Ignacio Horche Trueba, Beatriz Ramos Solano, F. Javier Gutiérrez Mañero

**Affiliations:** a Biobab R&D S.L. Calle Patones, s/n - Parcela 28.3 - P.I. Ventorro del Cano, (Madrid), 28925, Alcorcón, Spain; b Plant Physiology, Pharmaceutical and Health Sciences Department, Faculty of Pharmacy, Universidad San Pablo-CEU Universities, Boadilla del Monte, Spain

**Keywords:** New specie, Water stress, Induced systemic tolerance, Adaptative metabolism, Blueberry

## Abstract

A gram-positive, nonpathogenic, central endospore-forming, flagellated strain, was successfully isolated from the rhizosphere of *Pinus pinaster* in Aracena (Spain). Its optimal growth conditions are 28 °C, pH 6, and 0 % salinity. It is able to assimilate glucose, L-fucose, L-arabinose, b-metil-D-xylose and shows high catabolic capacity. The major fatty acids (>79.20 % of the total fatty acids) are anteiso C15:0 > iso C15:0 > C14:0.

A phylogenetic analysis based on the 16S rRNA gene sequence revealed similarity to *P. frigoritolerans* DSM8801^T^ (99.9 %), *P. castrilensis* CECT30509^T^ (99.8 %), and *P. simplex* DSM1321^T^ (99.6 %). Comparison of whole genomes revealed that strain BBB004^T^ is more similar to *P. simplex* DSM1321^T^. According to ANI (93.54 %), AAI (94 %), dDDH (60.6), %G + C (0.12), TETRA (0.99822) and intergenomic distance (0.2835) values, therefore this species is different to the closest. A total of 133 genes unique to *Peribacillus* BBB004^T^ were identified.

Supported by these analyses, strain BBB004^T^ (=LMG32742^T^ = CCUG76477^T^) was proposed as the type strain for a new species, named “*Peribacillus aracenensis* sp. nov.”

## 1 Introduction

Recently, an exhaustive analysis of *Bacillus* genomes, has resulted in the description of six new genera: *Peribacillus* gen. nov., *Cytobacillus* gen. nov., *Mesobacillus* gen. nov., *Neobacillus* gen. nov., *Metabacillus* gen. nov. and *Alkalihalobacillus* gen. nov., which allow a more adequate classification of the species previously ascribed to the genus *Bacillus* [1]. Patel and Gupta first described the genus *Peribacillus* in 2020 describing 15 validated species within this genus [2], among which some beneficial strains for agriculture are present.

*Pinus pinaster* is a fast-growing plant species from the *Pinaceae* family, native to the western Mediterranean region, that is well adapted to the hot and dry climate and nutrient-poor soils of the Mediterranean region. In Spain, *Pinus pinaster* is widely distributed along the Atlantic coast. The tree is commonly used for reforestation, as it can grow in a wide range of soil types and is resistant to drought and fire [3]. The wide distribution reveals its great innate adaptive capacity, for which the plant recruits the best strains to improve survival. Therefore, *Pinus pinaster* constitutes an excellent recruiter for beneficial strains to improve adaptation to drought and nutrient acquisition [4].

Plant growth promoting bacteria (PGPB) are a group of beneficial bacteria that can improve plant growth and health through various mechanisms. They colonize the root surface or rhizosphere, the soil surrounding the roots, and interact with the plant in a mutually beneficial manner [5]. PGPB can improve nutrient availability by solubilizing phosphate or producing siderophores that help plants acquire iron [4,6,7]. In addition, PGPB can protect plants from pathogenic bacteria and fungi by producing antibiotics and competing for resources. On the other hand, PGPB can modify plant metabolism by i) producing phytohormones such as auxins, gibberellins, and cytokinins, which stimulate plant growth and development by altering plant hormonal balance, or ii) they can also induce a systemic response to biotic stress, increasing resistance in plants, making them more resistant to future pathogen attacks, in a process known as Induced systemic resistance [8]; iii) PGPB can also trigger plant adaptation to abiotic stress in a process known as Induced systemic tolerance [9]. PGPB are an excellent tool to modulate the plant's ability to adapt to different stress situations by triggering adaptive mechanisms [9,10]. Overall, PGPB are an important tool for sustainable agriculture, as they can improve plant growth and health without the need for chemical fertilizers or pesticides and can help to reduce the amount of water required to grow crops, leading to more sustainable and efficient farming practices. Therefore, finding efficient isolates able to trigger plant physiology and adaptation is a challenging topic. For this purpose, scientist rely on the plant's ability to recruit effective strains, screening in the rhizosphere of wild species which are naturally adapted to harsh conditions [11].

As a component of an ongoing research endeavor, bacteria were isolated from the rhizosphere of *Pinus pinaster*, and the resultant strain, BBB004^T^, underwent comprehensive characterization. This investigation encompassed a thorough genetic scrutiny aimed at elucidating the phylogenetic positioning of strain BBB004^T^ within the *Peribacillus* genus. This was achieved by sequencing the entire genome and subsequently comparing it with the genomes most closely resembling it within the *Peribacillus* genus. A phenotypic and metabolic analysis was also conducted, followed by a biological assay to validate its potential applicability in agriculture, specifically in enhancing fruit yield within water-restricted conditions prevalent in blueberry-intensive orchards.

## 2 Results

### 2.1 Phenotypic characterization

Isolated from the rhizosphere of *Pinus pinaster*, strain BBB004^T^ was categorized as a gram-positive, nonpathogenic, flagellated (peritrichous flagellated rods; flagellum were sensitive to disruption of mucilagous capsule so length was not determined and showed an average width of 0,01-0,02 mm), central endospore-forming bacilli (5.95 μm × 1.34 μm) (Fig. 1A, B, C, &D, supplementary material). Its growth behavior on plate count agar (PCA) at 28 °C results in the formation of circular colonies with a diameter of less than 1 mm. These colonies exhibit rough boundaries, an opaque, white, and creamy texture. In liquid culture, particularly in nutrient broth, the strain displays a color transformation from pale yellow during the exponential growth phase to a vivid yellow during the stationary phase after being cultivated for 24 h at 28 °C.

The range of temperature-dependent growth at which *Peribacillus* BBB004^T^ grows ranges from 4 °C to 40 °C, although, it needs 72 h to grow at lower temperatures. In terms of pH tolerance, strain BBB004^T^ demonstrates growth within the pH spectrum of 5–7, while accommodating salt concentrations of up to 6 %. The most conducive conditions for the growth of BBB004^T^ are observed at an optimal temperature of 28 °C, a pH level of 6, and a salinity concentration of 0 %.

Metabolic capabilities were tested with API 50 CHB/E Medium & API20E (Tables 1A and 1B, supplementary material). API 50 CHB/E Medium shows its ability to assimilate several substrates namely, L-arabinose, b-methyl-D-xylose, glucose, and L-fucose was determined by evaluating the acid production capacity from each substrate. Compared to the closest strains *P. frigoritolerans* DSM8801^T^, *P. castrilensis* CECT30509^T^, *P. simplex* DSM1321^T^ and *P. muralis* DSM16288^T^, strain BBB004^T^ can degrade L-arabinose while *P. frigoritolerans* DSM8801^T^ and *P. castrilensis* CECT30509^T^ cannot; *b*-methyl-D-xyloside and L-fucose *P. frigoritolerans* DSM8801^T^*, P. simplex* DSM1321^T^*,* and *P. muralis* cannot, and glucose while *P. simplex* DSM1321^T^ and *P. castrilensis* CECT30509^T^ cannot; finally, *P. castrilensis* CECT30509^T^ can degrade adonitol and amygdaline while BBB0004^T^ cannot. API20E results positive for BBB004^T^ in urease, indole production, gelatin hydrolysis, and oxidase; comparing to the closest strains none of them resulted positive for urease, only *P. castrilensis* CECT30509^T^ for indole production, *P. castrilenis* CECT30509^T^, for gelatine hydrolysis and *P. simplex* DSM1321^T^ resulted variable, and *P. muralis* DSM16288^T^ for oxidase.

In addition, this strain resulted positive for auxins (2 ppm IAA-like), siderophore synthesis (3.5 mm diameter halo) and chitinases (3 mm diameter halo) production when tested *in vitro*. *P. simplex* DSM1321^T^, *P. muralis* DSM16288^T^, and *P. frigoritolerans* DSM8801^T^ resulted positive for auxins (under 2 ppm IAA-like), siderophore (2, 1.5 and 1 cm halos, respectively) and chitinases (1.2, 1.5 and 1 cm, respectively) synthesis, less intense than strain BBB004^T^ in all cases.

The predominant fatty acids of strain BBB004^T^ were anteiso C_15:0_ (51.6 %) and iso C_15:0_ (16.6 %) (table 2 supplementary material) similarly to the closest related type strains. The main difference with the closest strain *P. simplex* DSM1321^T^ was the higher contents on iso C_14:0_, and iso C_16:0_ that were 2-fold more abundant in BBB004^T^ than in *P. simplex* DSM1321^T^ (Table 2, supplementary material).

### 2.2 Phylogenetic analysis

The 16S rRNA gene, with a length of 1462 base pairs (accession number OP359306), served as a basis for analysis. Examination of the 16S rRNA gene revealed that strain BBB004^T^ exhibited the highest degree of similarity with *P. frigoritolerans* DSM8801^T^ (99.9 %), followed by *P. castrilensis* CECT30509^T^ (99.8 %), *P. simplex* DSM1321^T^ (99.6 %), *P. muralis* DSM16288^T^ (99.5 %), and *P. butanolivorans* DSM18926^T^ (99.4 %), (Table 3, supplementary material). To visually represent these relationships, a phylogenetic tree was constructed using the TYGS server (with the strains that reveals a similarity higher than 97 %), drawing upon the most closely aligned 16S rRNA gene sequences (Fig. 2 supplementary material).

The phylogenetic tree reveals three differentiated groups. Group 1 composed *by P. huizhouensis* GSS03^T^, *P. psychrosaccharolyticus* ATCC23296^T^, and *P. loiseleuriae* FJAT-27997^T^; Group 2 composed by *P. butanolivorans* DSM18926^T^, *P. simplex* DSM1321^T^, *P. frigoritolerans* DSM8801^T^, *P. castrilensis* CECT30509^T^, *P. muralis* DSM16288^T^, and BBB004^T^; Group 3 composed only by *P. asahii* MA001^T^. Based on the phylogenetic tree obtained comparing the 16S rRNA sequence, it seems that the closest related strain is *P. frigoritolerans* DSM8801^T^, but as we will see in the whole genome comparison it is very close but not the closest.

Among the genomes analyzed, the most notable ANI and AAI scores were recorded when comparing strain BBB004^T^'s genome to those of two species: *P. frigoritolerans* DSM8801^T^ (93.96 % ANI and 93.26 % AAI) and *P. simplex* DSM1321^T^ (93.54 % ANI and 94 % AAI). Notably, the most robust dDDH value of 60.6 % and the closest intergenomic distance of 0.2835 were also observed in the comparison between BBB004^T^ and *P. simplex* DSM1321^T^. This comparison exhibited a minor deviation of 0.12 % in G + C content between the genomes of these two strains. The range of TETRA values spanning from 0.91401 to 0.99822 indicates a lack of substantial genomic resemblance between strain BBB004^T^ and the other genomes (Table 1). *P. gossypii* JM-267^T^ could not be compared except for the 16S gene since there is not a whole genome sequence available.

Using the MASH algorithm, which provides a rapid estimation of intergenomic relatedness [12], the genome of BBB004^T^ was matched against all available type strain genomes within the TYGS server. Subsequently, the ten type strains exhibiting the closest MASH distances to the user genome were selected. This curated set of strains was then utilized to generate a phylogenetic tree (with the strains that reveals a similarity of 16S sequence higher than 97 %), depicted in Fig. 3 of supplementary material. he genome details for BBB004^T^ can be accessed at this link: https://www.ncbi.nlm.nih.gov/bioproject/PRJNA876065 (WGS: JANZMJ000000000).

The phylogenetic tree according to whole genome reveals three differentiated groups (Fig. 1). Group 1 composed *by P. asahii* MA001^T^*,* and *P. psychrosaccharolyticus* ATCC23296^T^; Group 2 composed by *P. loiseleuriae* FJAT-27997^T^*, P. huizhouensis* GSS03^T^; Group 3 composed by *P. butanolivorans* DSM18926^T^, *P. muralis* DSM16288^T^, *P. simplex* DSM1321^T^, *P. castrilensis* CECT30509^T^*, P. frigoritolerans* DSM8801^T^, and BBB004^T^. The phylogenetic tree obtained comparing the whole genomes sequences revealed that the closest related strain is *P. simplex* DSM1321^T^.

Genome mining of BBB004^T^ highlighted a number of genes traditionally related to plant growth promotion and plant fitness like auxin biosynthesis related genes (5), iron metabolism related genes (14), aromatic amino acid and derivatives related genes (44), exopolysaccharides biosynthesis related genes (6), and proline synthesis and uptake (11) (Table 4 supplementary material). Within these groups, some genes are important to denote. The subclass of “cell wall and capsule” was well represented. Within the “cofactors, vitamins, prosthetic groups, pigments”, menaquinone and phylloquinone biosynthesis related genes were observed (Table 4 supplementary material).

Upon conducting a comparative examination of the pangenome, it was revealed that BBB004^T^ uniquely possesses a collection of 133 genes (Table 5 supplementary material). Within this set of genes, a substantial proportion (25 %) was found to be linked with distinct subsystems, as illustrated in Fig. 2. The most represented subsystem was “fatty acids, lipids and isoprenoids” (15 unique genes), followed by “carbohydrates” (10 unique genes) and “protein metabolism” (7 unique genes) (Fig. 2).

### 2.3 Biological assay

Blueberries were harvested from February through May 2021, and a significant increase in total accumulated production (kg/plant) was recorded (+34 %) (Fig. 3, supplementary material).

Osmolytes significantly increased by an average 2-fold, both in proline (Fig. 4A, supplementary material) and soluble sugars (Fig. 4B, supplementary material).

Antioxidant polyphenols increased almost 4-fold (+272 %) (Fig. 5, supplementary material), associated with lower oxidative stress (−37 % MDA (Malondialdehyde) (Fig. 6, supplementary material), and a decrease in photosynthetic pigments chlorophyl a, chlorophyll *b* and carotenoids (40 %) (Fig. 7, supplementary material).

## 3 Discussion

The suitability of *Pinus* genera growing in dry and low nutrient soils to select effective strains for promoting plant growth and development by different mechanisms has been described before [4] and is confirmed in the present study. Strain BBB004^T^ has resulted positive for different *in vitro* PGPB characteristics related to nutrient mobilization (siderophores & phosphatases), to plant defense (chitinases) and to plant development (auxins), in view of which, this strain seemed like a putative good candidate for different applications in the agricultural area. This potential has shown in vivo under harsh conditions as it has been able to increase blueberry production under water limiting conditions, activating plant metabolism for a better adaptation.

To exploit its potential, a comprehensive multi-phase approach was adopted to delve into the genetic makeup of strain BBB004^T^ and uncover its concealed information. Initially, scrutiny of the 16S rRNA gene unveiled a remarkable resemblance, exceeding 99 %, to five species with validated names. The sequencing of the 16S ribosomal gene (16S rRNA) serves as a fundamental tool in the contemporary classification of bacterial species. Generally, instances where the 16S rRNA gene similarity falls below 98.65–99 % are acknowledged as indicators of distinct species [13]. However, the discriminatory capacity of the 16S rRNA gene can be limited, particularly for closely related species displaying nearly identical sequences (above 99 %), despite evident differentiation in terms of DDH values [14,15].

In line with this, ANI (average nucleotide identity) and AAI (average amino acid identity) values were harnessed to confirm discrepancies between BBB004^T^ and other validated genomes. These values serve as robust metrics for gauging evolutionary distance, as empirical evidence corroborates that ANI and AAI values of 95–96 % correspond to a DDH value (DNA-DNA hybridization) of 70 %, commonly utilized to delineate prokaryotic species [13,16,17]. The likeness between closely related strain genomes is reflected in similar tetranucleotide usage frequencies, with correlation indexes (TETRA) of 0.99 or higher [17,18]. The highest ANI and AAI values obtained during the comparison of BBB004^T^'s genome to species like *P. frigoritolerans* DSM8801^T^, *P. castrilensis* CECT30509 ^T^, *P. simplex* DSM1321^T^, *P. muralis* DSM16288^T^, and *P. butanolivorans* DSM18926^T^, all remained well below the 95 % threshold established for designating genomes as belonging to the same species. The most elevated dDDH value and the closest intergenomic distance were observed in the comparison between BBB004^T^ and *P. simplex* DSM1321^T^ (as shown in Table 1), with the dDDH value falling below the 70 % mark required to categorize BBB004^T^ under the same species. Despite the TETRA values suggesting similarities between BBB004^T^'s genome and those of *P. frigoritolerans* DSM8801^T^, *P. castrilensis* CECT30509 ^T^ or *P. simplex* DSM 1321^T^ (0.99704, 0.99777, and 0.99822 % respectively), other indicators convincingly disprove any association between BBB004^T^ and these species.

**Table 1:** Similarity of the 10 genomes with strain BBB004^T^ genome.

**Rank**	**Name**	**Genome Accession number**	**16S(%)**	**ANI(%)**	**dDDH(%)**	**Differences in G + C content**	**TETRA(%)**	**AAI(%)**	**Intergenomic distance**
1	*Peribacillus simplex* DSM1321^T^	BCVO01000086	99.6	93.5	60.6	0.1	0.99	94	0.2
2	*Peribacillus frigoritolerans* DSM8801^T^	AM747813	99.9	93.9	59.2	0.6	0.99	93.2	0.2
3	*Peribacillus castrilensis* CECT30509^T^	JAJNAF010000000	99.8	93.0	59.3	0.2	0.99	93	0.2
4	*Peribacillus muralis* DSM16288^T^	LMBV01000055	99.5	86.4	31.9	0.9	0.98	86.5	0.5
5	*Peribacillus butanolivorans* DSM18926^T^	LGYA01000001	99.4	86.5	34.2	2.1	0.95	86.9	0.5
6	*Peribacillus loiseleuriae* FJAT27997^T^	LFZW01000001	97.6	86.5	13.8	2.4	0.92	67.4	0.9
7	*Peribacillus gossypii* JM267^T^	KT240114	97.5	X	X	X	X	X	X
8	*Peribacillus asahii* MA001^T^	QWVS01000027	97.3	85.4	14.4	2.4	0.91	70.7	0.9
9	*Peribacillus psychrosaccharolyticus* ATCC23296^T^	AB021195	97.1	85.6	13.8	1.1	0.92	67.8	0.9
10	*Peribacillus huizhouensis* FJAT 14515^T^	KJ464756	97.0	86.8	13.8	2.9	0.91	67.6	0.9

In the phylogenetic tree constructed using complete genome data in TYGS (Fig. 1), BBB004^T^ exhibits a close relationship with *P. simplex* DSM 1321^T^. While it has been documented that *Peribacillus* species can ameliorate drought stress [19], this comprehensive genomic analysis underscores BBB004^T^'s classification as a novel species with beneficial agricultural attributes which is strongly supported by the biological assay.

**Fig. 1:**
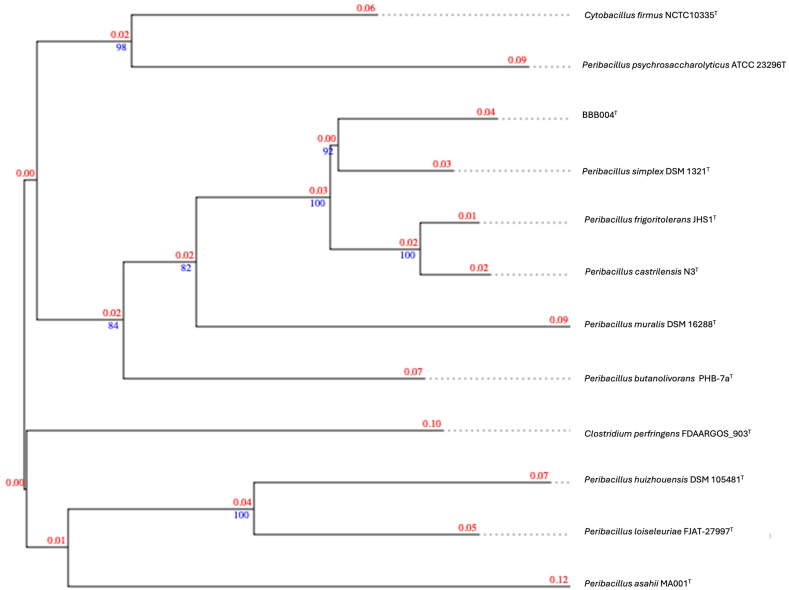
Phylogenetic tree constructed in TYGS. Tree inferred with FastME 2.1.6.1 [20] from GBDP distances calculated from genome sequences. The branch lengths are scaled in terms of GBDP distance formula d5. The numbers above the branches are GBDP pseudo-bootstrap support values > 60 % from 100 replications, with an average branch support of 94.3 %. The branch length values represent the evolutionary time between two nodes. Unit: substitutions per sequence site [21].

**Fig. 2:**
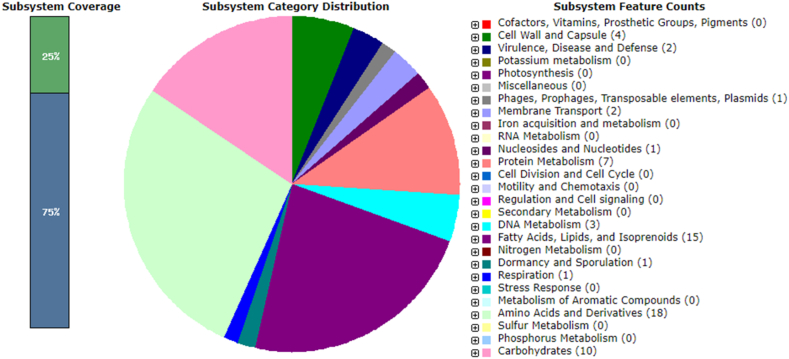
Representation of functional categories of novel regions of strain BBB004^T^.

Analysis of whole genome revealed menaquinone and flagellum related genes in BBB004^T^. We anticipate that no differences in menaquinone composition to other species within this genus will be found, consistent with other authors who stablish differences in menaquinone profiles only at genus level [22].

The 133 unique genes of this strain suggest an extraordinary ability to metabolize lipids, carbohydrates and proteins, indicating that this strain has a versatile and more competitive metabolism in the rhizosphere.

**figs4:**
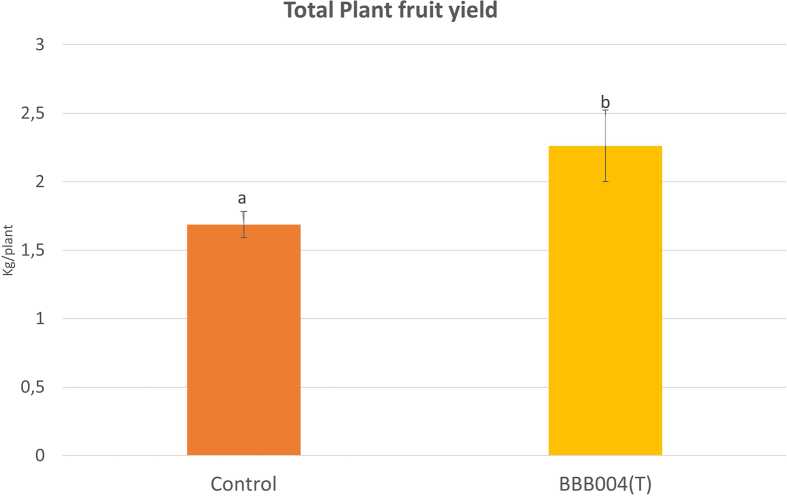


**figs5:**
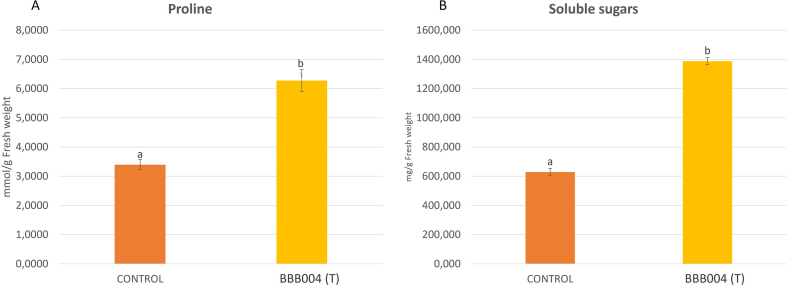


**figs6:**
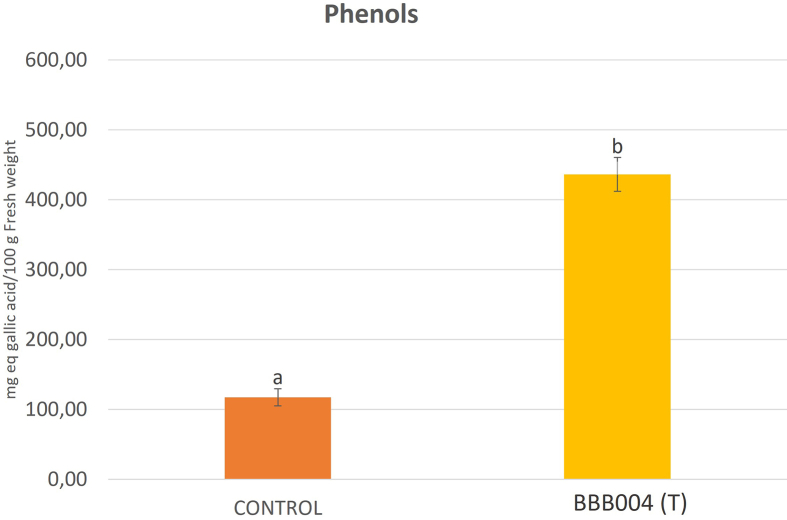


**figs7:**
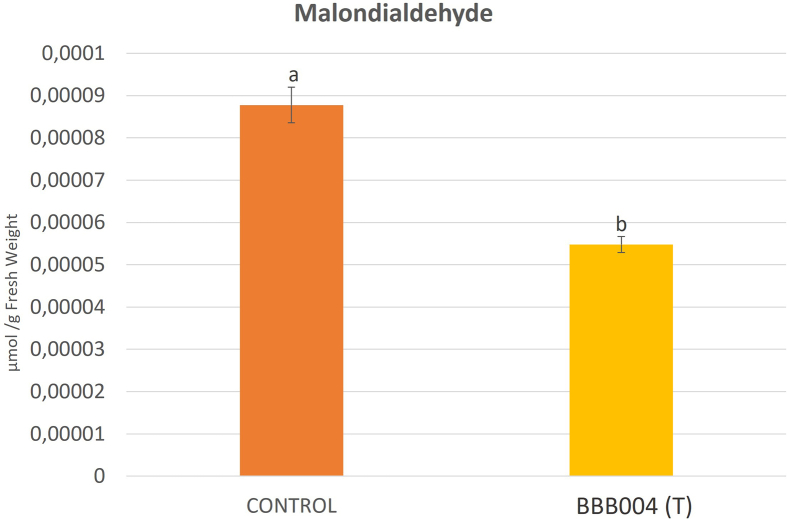


The biological assay has demonstrated the potential of this strain to increase production by 34 % (Fig. 2, supplementary material) with a reduction of irrigation volume along the plant cycle in field conditions. As blueberry production is considered a hydroponic system because all nutrients are delivered through irrigation, nutrients were also limited to treated plants, ruling out the possibility of providing more nutrient to treated plants than to controls. Among the underlying reasons for this yield improvement are the metabolic changes that allow a better hydration of plants, as suggested by the increase of osmolytes observed (Fig. 4, supplementary material) [23,24]. On the other hand, BBB004^T^ contributes to lower oxidative stress in plants as indicated by lower MDA concentration in leaves (Fig. 6, supplementary material). Among the possible reasons for this physiological status are the decrease of photosynthetic pigments (Fig. 7, supplementary material), that will be responsible for a more efficient partitioning of absorbed energy and therefore, a more efficient photosynthesis [25], and also, to lower physiological ROS production due to photosynthesis [26,27]. Also contributing to the lower oxidative stress is the amazing increase in phenols with a strong antioxidant role (Fig. 5, supplementary material) that contribute to balance ROS homeostasis. This strain shows good potential for agriculture under harsh conditions, in line with the UN goals 12 (responsible production and consumption) to achieve zero hunger (goal 2).

To consolidate, the culmination of both phenotypic and genomic investigations unequivocally establishes its unique and unmistakable genetic makeup, clearly distinguishing it from all established *Peribacillus* species with documented reference genomes. Hence, grounded in this comprehensive and polyphasic methodology the following description is made.

### 3.1 Description of *Peribacillus aracenensis* sp. nov

*“Peribacillus aracenensis* (aracen.en′sis), N.L. masc. adj. aracenensis, belonging to Sierra de Aracena, Huelva, Spain, from which the type strain was isolated”.

Cells are Gram-reaction positive, aerobic, rod-shaped bacterium ranging between 1.34 μm wide x 5.95 μm long, flagellated endospore-forming rods. Colonies grow on plate count agar (PCA) at 28 °C forming circular colonies < 1 mm ∅, with smooth borders, opaque, whitish, and creamy texture. In liquid culture (NB, Nutrient broth), color changes from pale yellow in the exponential growth phase to intense yellow on the stationary phase after 24 h at 28 °C. Growth occurs from 22 to 40 °C in 24 h, but not over 40 °C. As regards to pH, strain BBB004^T^ grows in a pH range from 5 to 7, loosing viability at pH 4 or 8, and with a salt concentration from 2 to 6 %. The optimal temperature, pH and salinity of growth for BBB004^T^ is 28 °C, pH 6, and 0 % salinity. It is able to assimilate glucose, L-fucose, L-arabinose, β-metil-D-xylose. It is able to produce auxins, siderophores and chitinases. The major fatty acids (>79.20 % of the total fatty acids) are anteiso C_15:0_ > iso C_15:0_ > C_14:0_.

Strain BBB004^T^ was isolated from Sierra de Aracena region in Huelva, Spain, in 2019. We introduce a previously unidentified *Peribacillus* species, denoted as *Peribacillus aracenensis* sp. nov., with a G + C content of 40.01 %. The draft genome and 16s rDNA sequence are deposited at NCBI WGS JANZMJ000000000 and OP359306, respectively. The type strain is BBB004^T^ (=LMG32742 ^T^ = CUGG76477 ^T^).

## 4 Materials and methods

### 4.1 Origin of bacteria

In October 2019, bacterial screening was conducted within the rhizosphere of wild *Pinus pinaster* populations situated in Sierra de Aracena, Sevilla, Spain (coordinates UTM 6°35W 37°55N). The soil intimately adhering to the roots and the finer roots (with a diameter of 1–2 mm) from four distinct plants were randomly amalgamated to form a single sample, with a total of four such replicates being sampled. All collected materials were securely contained within plastic bags and maintained at a temperature of 4 °C during transportation to the laboratory. One gram of rhizosphere soil, along with the finer roots, was subsequently mixed in a homogenizer with 10 mL of sterile distilled water for a duration of 1 min.

Following homogenization, a portion of the soil suspension, specifically 100 μL, was employed to generate serial 10-fold dilutions within a final volume of 1 mL. From each serial-dilution series, which was based on each replicate (totaling 4 replicates), 100 μL were cultured on Standard Methods Agar (Pronadisa SPAIN) and then incubated at 28 °C for a period of 4 days. After 36 h of incubation, individual colonies were meticulously chosen and marked on the agar plate to prevent duplication.

From every serial-dilution series, a sum of 80 colony forming units (cfu) were meticulously selected, resulting in a total of 320 cfu when accounting for all four replicates. These isolated units were further categorized into four parataxonomic groups based on distinct Gram staining [28], morphological characteristics, and sporulation capabilities. These groups were defined as follows: Gram-positive endospore-forming bacilli, Gram-positive non-endospore-forming bacilli, Gram-negative bacilli, and any other morphologies were collectively grouped as "Others."

Subsequently, all the isolated specimens were meticulously preserved at a temperature of −20 °C using a solution of glycerol and water in a 1:4 ratio, as described previously [11,29]. The bacterium of interest (*P. aracenensis* BBB004^T^) was isolated from these dilutions and maintained at −20 °C using a glycerol-water mixture in a 1:4 ratio.

### 4.2 Phenotypic characterization

We subjected our bacterium to a battery of *in vitro* tests to evaluate the production of auxins, siderophores and chitinases [30]. Auxins were evaluated according to Ref. [31] on the supernatant of a 24h bacterial culture free of bacteria, by adding Salkowski reagent, and measuring absorbance at 525 nm after 25 min; data was interpolated on IAA calibration curve; a positive result was considered from 2 ppm IAA-like. The potential of the closest related strains was also evaluated for all *in vitro* tests.

Siderophores were determined according to Ref. [32] on CAS-agar plates by the presence of orange halos after 48h, indicative of siderophore presence; bacterial siderophores bind the Fe-complexed on CAS, changing the color from blue to orange. A positive result was considered for halos over 2 mm diameter.

Chitinase production was determined according to Ref. [30] on agar plates loaded with insoluble colloidal chitin, after 7 days of incubation; presence of a transparent halo over 2 mm is indicative of the presence of chitinases.

Firstly, morphology of colonies was assessed on Plate count agar (PCA) (BAC ISO-4833 labkern)) medium at 28 °C after 24 h, while other phenotypic traits were done in Nutrient Broth (Condalab). Additionally, Gram staining [28] was performed using established procedures.

In order to characterize bacterial growth under various temperatures (4, 22, 28, 30, 37, 40, 50 °C), pH (4, 5, 6, 7, 8, 9, 10), and salt concentration (2, 4, 6, 7, 10 %) conditions, the following protocols were implemented. For each condition, culture media was loaded on 10 mL tubes of the specific medium, and after inoculation, tubes were inoculated at the desired condition for 48h with continuous shaking at 140 revolutions per minute (rpm) using an orbital shaker. In all cases at 24h preinoculum optical density 600 nm = 1, delivered at 1 % was used. A SPECTROstar nano (BMG Labtech) was used to measure OD. Cell viability was established relating OD to cell growth by the drop method [33].

Temperature: The spectrum of temperature tolerance was explored at seven different temperatures (4C, 22C, 28C, 30, 37C, 40C, and 50). To determine growth at 4 °C, a plate was incubated for 1 week at 4C in a regular refrigerator; all other conditions were evaluated on plates, as described above. OD was measured and cell density was established.

pH: The spectrum of pH ranged from 4 to 10 at 1 pH unit intervals. Growth tests were conducted on nutrient broth adjusting pH with appropriate buffer systems: pH 4–5.5 with 0.1 M citric acid/0.1 M sodium citrate; pH 6–9 with 0.1 M KH_2_PO_4_/0.1 M NaOH; and pH 8.5–10 with 0.1 M NaHCO_3_/0.1 M Na_2_CO_3._ The medium's pH was adjusted post-autoclaving by addition of 1 M NaOH/HCl before sterile filtration through a 0.22 μm pore size filter. After incubation for 24h under shaking, OD was measured, and cell density was established.

Salt tolerance. Growth tests were conducted in the presence of NaCl ranging from 0 to 8 % (wt/vol) at 1 % intervals. After incubation, OD was measured, and cell density stablished.

Lastly, the metabolic capabilities were evaluated using the API 50 CHB/E Medium & API20E in accordance with manufacturer instructions. This test was performed in triplicate. All strains were evaluated in parallel, except for *P.castrilensis* CECT30509^T^, for which data was taken from Ref. [2].

The cellular Fatty Acid composition was determined following the protocol recommended by MIDI Microbial Identification System [34]. The cellular content of Fatty Acids was obtained using an Agilent 6850 gas chromatograph, with the MIDI Microbial Identification System using the TSBA6 method (MIDI 2008).

The culture conditions of strain BBB004^T^ until the biomass was obtained were nutrient broth, 28 °C, 24 h incubation. The fatty acid profile obtained is shown below, as well as its relative composition in % and the corresponding chromatogram.

### 4.3 Phylogenetic analysis

The genomic analysis of strain BBB004^T^ involved utilizing the resources offered by EzBioCloud. Initially, EzBioCloud's identification feature was employed to conduct similarity-based searches against meticulously curated databases housing 16S rRNA sequences. Subsequently, a comprehensive assessment encompassing Average Nucleotide Identity (ANI), Average Amino Acid Identity (AAI), digital DNA-DNA hybridization (dDDH), and TETRA analysis was carried out. For a thorough examination of the entire genome, the Type Strain Genome Server (TYGS) was employed.

Before any analysis was conducted, possible contamination was removed by PhiX phage (commonly used as a calibration control in Illumina sequencing) with BBDuk and duplicate reads using the clumplify.sh script, both tools included in the BBmap 38.90 package [35]. Next, the Trimmomatic v0.39 was used to remove the sequencing adaptors (ILLUMINACLIP option) and low-quality regions of the reads (TRAILING:10, LEADING:10, HEADCROP:12 and SLIDINGWINDOW:5:30). Finally, BMTagger was also applied to identify and remove any possible human readout present in the dataset.

To explore the taxonomic positioning of strain BBB004^T^ and its phylogenetic relationship with other species, we employed three primary methodologies.

1. Genomic Similarity Estimation: a) Utilizing a phylogenetic analysis and relying on the examination of the 16S rRNA, we endeavored to delineate species boundaries. b) We assessed the average nucleotide identity (ANI), average amino acid identity (AAI), digital DNA-DNA hybridization (dDDH), differences in guanine-cytosine (G + C) content, and the frequency of tetranucleotides (TETRA) use to gauge genomic likeness.
2. Phylogenetic Analysis at the Whole Genome Level: By conducting a comprehensive phylogenetic analysis using complete genome sequences, we aimed to discern the broader evolutionary context of strain BBB004^T^.
3. Identification of Novel Genomic Regions: Our exploration encompassed the identification of novel genetic regions that could potentially contribute to the distinct characteristics of strain BBB004^T^.

The 16S rRNA gene, with a length of 1462 base pairs was obtained by PCR using 27F and 1492R primers, and it is sequenced using 785F and 907R primers [36], which are the inter-primers, for identification.

The complete genome sequencing was executed on the Illumina platform MiSeq PE300, generating a substantial collection of 1,259,190 sequences. These sequences underwent rigorous quality filters and were subsequently assembled de novo to construct longer sequences, known as scaffolds. This process led to the generation of 129 scaffolds, collectively comprising a total length of 5.9 Mb for the *Peribacillus* BBB004^T^ genome.

Further validating this assembly, mapping the sequences back onto the assembled genome confirmed that a noteworthy proportion, specifically 92.63 %, of the sequences derived from *Peribacillus* BBB004^T^ were successfully incorporated into the assembly process.

The 16S rRNA BBB004^T^ sequence obtained by PCR were subjected to BLAST queries against the complete set of strains with validated names within the reference database, available on the Ezbiocloud website.

The first stage of this analysis consists in the comparison at the nucleotide (ANI) and amino acid (AAI) level of *Peribacillus* BBB004^T^ genome with all complete genomes available in the EzBiocloud database for similar genomes according to the 16s analysis previously performed. The estimates of the nucleotide identity index (ANI) were calculated using (https://www.ezbiocloud.net/tools/ani). The amino acid content (AAI) was calculated at http://enve-omics.ce.gatech.edu/aai/. For ANI and AAI, values below 95 % indicate a new species [13,17]. Building upon the outcomes of the preceding section, a subset of 27 genomes situated within the fourth quartile of the ANI values distribution was singled out. This selection strategy, by design, targeted those genomes anticipated to be the closest relatives of *Peribacillus* BBB004^T^. For these chosen genomes, we proceeded to compute the intergenomic distances and dDDH (digital DNA-DNA hybridization) indices utilizing the GGDC web server (Genome-to-Genome Distance Calculator). The GGDC web server additionally facilitates the assessment of disparities in the G + C content across the genomes under scrutiny. It's crucial to note that values falling below the 70 % threshold are indicative of noteworthy dissimilarities, as established by previous studies [13,17].

For the determination of dissimilarities in tetranucleotide usage profiles between the genome of *Peribacillus* BBB004^T^ and its 27 closest counterparts, we employed the JSpecies software [17]. Notably, values surpassing the 99 % threshold are strong indicators of noteworthy distinctions, as elucidated by previous studies [17,18].

The genome sequence of BB004^T^ was submitted to the Type (Strain) Genome Server (TYGS), an accessible bioinformatics platform accessible at https://tygs.dsmz.de, for a comprehensive taxonomic analysis centered around whole-genome data [37].

Initially, the BB004^T^ genome underwent comparison against all available type strain genomes within the TYGS server using the MASH algorithm—a rapid approximation of intergenomic relatedness [12]. From this analysis, the ten type strains exhibiting the closest MASH distances to the user genome were identified. This preliminary selection was employed to identify the top 50 matching type strains per user genome based on bitscore. Subsequently, exact distances were computed utilizing the Genome BLAST Distance Phylogeny (GBDP) method with the 'coverage' algorithm and the d5 distance formula [37]. These distances were then utilized to determine the ten closest type strain genomes for each user genome.

In terms of phylogenomic inference, the set of genomes underwent pairwise comparisons via GBDP, which yielded precise intergenomic distances. This process employed the 'trimming' algorithm and the d5 distance formula [38], and 100 distance replicates were computed for each analysis. The computation of digital DNA-DNA hybridization (dDDH) values and their confidence intervals was carried out using the established settings of the GGDC 2.1 [37].

The resulting intergenomic distances were leveraged to construct a well-informed minimum evolution tree with balanced branches, employing FASTME 2.1.6.1 along with SPR postprocessing [20]. The branch support was gauged through 100 pseudo-bootstrap replicates. The trees were rooted at the midpoint, as proposed by Ref. [21], and visualized using PhyD3 [39].

The identification of novel regions was accomplished using the Pan-seq web server (https://lfz.corefacility.ca/panseq/page/index.html). The comparisons leading to this determination were facilitated through the application of the nucmer program from MUMmer v3. For added context, the annotation of these novel sequences was executed utilizing eggnog-mapper [38], which relied on eggNOG 4.5 orthology data [40].

### 4.4 Biological assay, irrigation reduction on blueberry experimental set up

This experiment was conducted on a two-year-old intensive blueberry (*Vaccinium corymbosum* var cupla) plantation located in Huelva, Spain. Within this plantation, two lines each spanning 60 m were meticulously chosen. Treatments were implemented within these two lines according to a randomized block design. Specifically, the treatments were replicated three times, each consisting of seven plants. The BBB004^T^ treatment encompassed the inoculation of plant roots (75 mL per plant, with a concentration of 1 × 10^8^ colony-forming units/mL) every two weeks, commencing from October and extending up to May. On the other hand, the control group was subjected to mock inoculation using water, resulting in a total of 16 inoculation occurrences.

To induce a 25 % reduction in irrigation water, adjustments were made to the irrigation system by reducing the number of drippers per meter. This alteration in irrigation was aimed at achieving the targeted water reduction of 25 %. All nutrients are delivered through the irrigation system so there was a reduction in the provided nutrients as compared to controls. The amount of nutrients provided with inoculum accounted for 75 mL of culture media that was already diluted 1/10, so 7.5 mL of culture media every 15 days provided 5 mg of C and 5 mg of N per plant [41].

The assessment of production (kg/plant) was determined by dividing the accumulated fruit yield across the 21 plants per treatment by the total number of plants. The sampling point was strategically set in September, following the period of summer-induced stress. During this sampling, a comprehensive evaluation was carried out, encompassing various markers. These markers included photosynthetic pigments, malondialdehyde (MDA) as an indicator of cellular oxidative stress, phenolic compounds serving as antioxidants, and osmolytes such as proline and soluble sugars, all of which were subject to thorough analysis.

Extraction and quantification of pigments was done according to Ref. [42] with the formulae indicated by Ref. [43]. In short, powdered leaves (100 mg) were extracted overnight at 4 °C with acetone 80 % (v/v, 1 mL), centrifuged and absorbance determined at 647, 663, and 470 nm on a Biomate 5 spectrophotometer. The formulae used were.

• Chl a (mg/g FW) = [ (12.25xAbs663) - (2,79 x Abs647) ] x V(ml)/weigh(mg).
• Chl b (mg/g FW) = [ (21,5 x Abs647) - (5,1 x Abs663) ] x V(ml)/weigh(mg).
• Carotenoids (mg/g FW) = [ ((1000 x Abs470) - (1,82 x Chl a) - (85,02 x Chl b))/198 ] x V(ml)/weigh(mg).

For total phenols (Tubes were protected from light throughout the whole process) methanolic (80 %) leaf extracts were prepared by sonicating material for 10 min followed by centrifugation for 5 min at 5000 rpm. Then total phenols were quantified with Folin-Ciocalteu agent (Sigma. Aldrich, St Louis, MO) as described in Ref. [44], with modifications as in Ref. [27]. Results are expressed in mg of gallic acid equivalents per 100 g of fresh weight (FW).

For proline and soluble sugars an ethanolic extract (70 % ethanol (*v/v*)) was prepared with 0.25 g of leaf powder in 5 mL ethanol, incubated at 100 °C for 20 min and kept at 4 °C until analysis of proline and soluble sugars. For proline determination one mL of freshly prepared ninhydrin reagent (1 g of ninhydrin in 60 mL glacial acetic acid, 20 mL ethanol, 20 mL water) was mixed with 500 mL of extract, vortexed and heated for 20 min at 95 °C. After cooling, absorbance at 520 nm was determined. Results are expressed as μmol g ^−1^ [45].

Soluble sugars were determined according to Ref. [46] by incubating antrone reagent (3 mL) and 100 mL of the plant extract at 100 °C for 10 min. After cooling, absorbance was determined at 620 nm. Soluble sugar concentration was calculated with the following equation:

$$\mu g.\;g^{-1}=\left[\left(\text{Abs}620\;-0.016\right)/0.02\right]/\left(g\right)/1000$$

Malondialdehyde (MDA) was determined as in Ref. [47]. Leaf powder (100 mg) was suspended in 2 mL 10 % trichloroacetic acid and thoroughly vortexed for 2–3 min, followed by centrifugation at 20,000×*g* for 30 min at 4 °C. One mL of the supernatant was mixed with 4 mL 0.5 % (v/v) thiobarbituric acid (TBA) and 20 % (*v/v*) trichloroacetic acid (TCA), heated at 95 °C for 30 min and the reaction was stopped on ice. After 10 min centrifugation, absorbance was determined 532 and 600 nm. To calculate MDA content the following formula was used: MDA (nmol g FW^−1^) = [(OD532-OD600)]/(ε x FW), where FW is the fresh weight and ε the extinction coefficient (155 mM^−1^cm^−1^). Data were expressed as μmol g FW^−1^ (fresh weight).

### 4.5 Statistical analysis

To evaluate treatment effects on all variables, one-way ANOVA analysis was performed. When significant differences appeared (p < 0.05), LSD test (Least Significant Difference) Fisher was used. Statgraphics Centurion XVIII for Windows was used.

## CRediT authorship contribution statement

**Enrique Gutierrez-Albanchez:** Writing – original draft, Formal analysis, Data curation. **Ana García-Villaraco:** Writing – review & editing, Methodology, Investigation. **José Antonio Lucas:** Writing – review & editing, Investigation. **Ignacio Horche Trueba:** Funding acquisition. **Beatriz Ramos Solano:** Writing – review & editing, Supervision, Conceptualization. **F. Javier Gutiérrez Mañero:** Writing – review & editing, Investigation, Conceptualization.

## Data availability statement

The datasets presented in this study can be found in online repositories. The names of the repository/repositories and accession number(s) can be found below: https://www.ncbi.nlm.nih.gov/bioproject/?term=bbb004.

## Declaration of competing interest

The authors declare that they have no known competing financial interests or personal relationships that could have appeared to influence the work reported in this paper.

## Figures and Tables

**figs1:**
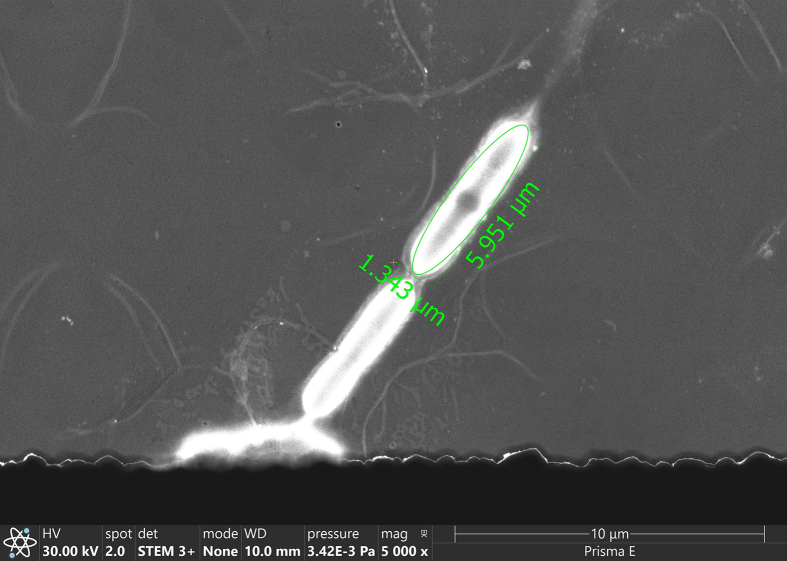


**figs2:**
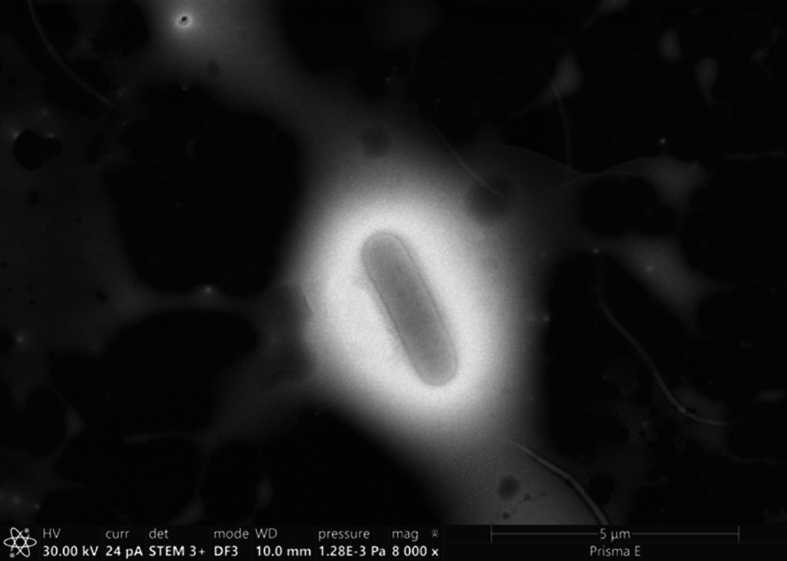


**figs3:**
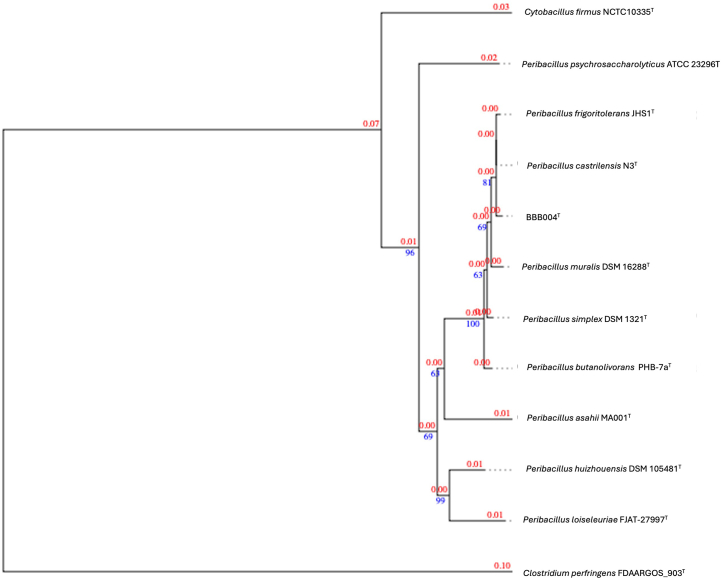


**figs8:**