# Supplementary material for: Peribacillus aracenensis sp.nov., a plant growth promoting bacteria for agriculture in water-scarce conditions isolated from Pinus pinaster rhizosphere
Source: Heliyon. 2024 Nov 5;10(22):e39973. doi: 10.1016/j.heliyon.2024.e39973 (PMC11583696; doi:10.1016/j.heliyon.2024.e39973)
Supplement: Multimedia component 1 [file mmc1.docx]

**Figure 1A&B.** *Peribacillus aracenensis* electronic microscope photograph.

**Figure 2.** Tree inferred with FastME 2.1.6.1 [13] from GBDP distances calculated from 16s rRNA gene sequences. The branch lengths are scaled in terms of GBDP distance formula d5. The blue numbers are GBDP pseudo-bootstrap support values > 60% from 100 replications, with an average branch support of 80.9%. The branch length values (in red) represent the evolutionary time between two nodes. Unit: substitutions per sequence site. The tree was rooted at the midpoint [14].

**Figure 3.** Total Plant fruit yield (kg/plant) in blueberry plants under limited watering (25% reduction) in controls and BBB004 ^T^ - inoculated plants (n=3).

**Figure 4.** Osmolytes. Leaf contents in Proline (mmol/g Fresh weight) and soluble sugars (mg/g Fresh weight) in controls and BBB004 ^T^ - inoculated plants (n=3). Different letters indicate significant differences according to LSD (Least significant difference) (p<0.05)

**Figure 5**. Total Phenol contents in leaves (mg eq gallic acid/100 g Fresh weight) of controls and BBB004 ^T^ -inoculated plants (n=3). Different letters indicate significant differences according to LSD (p<0.05)

**Figure 6.** Total malondialdehyde contents (μmol /g FW) (MDA) in leaves of controls and BBB004 ^T^ - inoculated plants (n=3). Different letters indicate significant differences according to LSD (p<0.05)

**Figure 7.** Photosynthetic pigments contents ((mg/g Fresh weight): A) chlorophyll a, B) chlorophyll b, C) carotenoids in leaves of controls and BBB004 ^T^ - inoculated plants under water limiting conditions (n=3). Different letters indicate significant differences according to LSD (p<0.05).
