# Supplementary material for: Peribacillus aracenensis sp.nov., a plant growth promoting bacteria for agriculture in water-scarce conditions isolated from Pinus pinaster rhizosphere
Source: Heliyon. 2024 Nov 5;10(22):e39973. doi: 10.1016/j.heliyon.2024.e39973 (PMC11583696; doi:10.1016/j.heliyon.2024.e39973)
Supplement: Multimedia component 2 [file mmc2.docx]

**Table 1**. API50-acid production capacity. Metabolic characteristics that differentiate strain BBB004^T^ from the type strains of the closest related *Peribacillus* species. Nd= non determined (n=3)*.* Data of *P. castrilensis* CECT30509^T^ was obtained from [13].
