# Supplementary material for: Peribacillus aracenensis sp.nov., a plant growth promoting bacteria for agriculture in water-scarce conditions isolated from Pinus pinaster rhizosphere
Source: Heliyon. 2024 Nov 5;10(22):e39973. doi: 10.1016/j.heliyon.2024.e39973 (PMC11583696; doi:10.1016/j.heliyon.2024.e39973)
Supplement: Multimedia component 3 [file mmc3.docx]

**Table 2.** Cellular fatty acid content of BBB004^T^ and related species of the *Peribacillus* genus. Data for *P.castrilensis* was obtained from Rodríguez González et al., 2022.. ND: non detectable. Summed feature* represents two or three fatty acids that cannot be separated using the MIDI system. Summed feature comprises iso C _17:1_, anteiso C _17:_ B.
