# Supplementary material for: Peribacillus aracenensis sp.nov., a plant growth promoting bacteria for agriculture in water-scarce conditions isolated from Pinus pinaster rhizosphere
Source: Heliyon. 2024 Nov 5;10(22):e39973. doi: 10.1016/j.heliyon.2024.e39973 (PMC11583696; doi:10.1016/j.heliyon.2024.e39973)
Supplement: Multimedia component 4 [file mmc4.docx]

Table 3. Similarity of the 16S rRNA of the 10 taxa with valid names and strain BBB004^T^.
